# Profiling of proteolytic enzymes in the gut of the tick *Ixodes ricinus *reveals an evolutionarily conserved network of aspartic and cysteine peptidases

**DOI:** 10.1186/1756-3305-1-7

**Published:** 2008-03-18

**Authors:** Daniel Sojka, Zdeněk Franta, Martin Horn, Ondřej Hajdušek, Conor R Caffrey, Michael Mareš, Petr Kopáček

**Affiliations:** 1 Institute of Parasitology, Biology Centre, Academy of Sciences of the Czech Republic, České Budějovice, CZ-370 05, The Czech Republic; 2 Institute of Organic Chemistry and Biochemistry, Academy of Sciences of the Czech Republic, Praha, CZ-166 10, The Czech Republic; 3 Faculty of Science, University of South Bohemia, České Budějovice, CZ-370 05, The Czech Republic; 4 Sandler Center for Basic Research in Parasitic Diseases, University of California San Francisco, San Francisco, CA 94158, USA

## Abstract

**Background:**

Ticks are vectors for a variety of viral, bacterial and parasitic diseases in human and domestic animals. To survive and reproduce ticks feed on host blood, yet our understanding of the intestinal proteolytic machinery used to derive absorbable nutrients from the blood meal is poor. Intestinal digestive processes are limiting factors for pathogen transmission since the tick gut presents the primary site of infection. Moreover, digestive enzymes may find practical application as anti-tick vaccine targets.

**Results:**

Using the hard tick, *Ixodes ricinus*, we performed a functional activity scan of the peptidase complement in gut tissue extracts that demonstrated the presence of five types of peptidases of the cysteine and aspartic classes. We followed up with genetic screens of gut-derived cDNA to identify and clone genes encoding the cysteine peptidases cathepsins B, L and C, an asparaginyl endopeptidase (legumain), and the aspartic peptidase, cathepsin D. By RT-PCR, expression of asparaginyl endopeptidase and cathepsins B and D was restricted to gut tissue and to those developmental stages feeding on blood.

**Conclusion:**

Overall, our results demonstrate the presence of a network of cysteine and aspartic peptidases that conceivably operates to digest host blood proteins in a concerted manner. Significantly, the peptidase components of this digestive network are orthologous to those described in other parasites, including nematodes and flatworms. Accordingly, the present data and those available for other tick species support the notion of an evolutionary conservation of a cysteine/aspartic peptidase system for digestion that includes ticks, but differs from that of insects relying on serine peptidases.

## Background

Ticks are important vectors of infectious agents causing diseases in human and domestic animals [1]. The castor bean tick *Ixodes ricinus *transmits Lyme disease caused by *Borrelia burgdorferi *spirochetes and tick borne encephalitis caused by the tick-borne encephalitis virus [2].

Blood-feeding and -digestion are essential activities for ticks. Blood provides a rich source of proteins and nutrients for anabolic processes such as vitellogenesis and egg production [3]. Unlike other blood-feeding arthropods, ticks are believed to digest blood intracellularly – in the endo/lysosomal vesicles of gut cells [4] at pH values well below the pH 6.3 – 6.5 of the gut contents [5,6]. Digestive gut cells use both receptor-mediated and fluid-phase endocytoses to uptake the liquid blood meal from the gut lumen [7]. Lara et al. [8] showed that the digestive cells of *Boophilus microplus *have separated endocytic pathways for two major proteins of host blood – serum albumin and hemoglobin. The requirement for receptor-mediated endocytosis might be directly linked to the detoxification of released heme groups during intracellular digestion of hemoglobin. Most of the toxic heme forms a unique type of heme aggregate ultimately accumulated inside specialized organelles called hemosomes [9]. The virtual absence of extracellular digestive enzymes in ticks enables the gut lumen to serve as a major storage organ [4].

In spite of the above studies, our understanding of the molecular proteolytic machinery involved in digesting host proteins in the tick gut is still rather fragmented. Previous studies have tended to focus on individual enzymes in particular species; all however, have identified either cysteine or aspartic peptidases; e.g., a cysteine class cathepsin L in *B. microplus *[10], two forms of a cathepsin L in *Haemaphysalis longicornis *[11] and the aspartic peptidase, cathepsin D (termed longepsin) in *H. longicornis *[12]. Also, cysteine-class asparaginyl endopeptidases (AE, legumains) have been characterized in *I. ricinus *[13] and *H. longicornis *[14].

The data thus far from different tick species raise the hypothesis that tick intestinal digestion relies on an evolutionarily conserved network of cysteine and aspartic peptidases characterized in other parasites, including platyhelminths [15,16] and nematodes [17]. It comprises mainly cysteine peptidases cathepsin B, L, C, asparaginyl endopeptidase/legumain and an aspartic peptidase cathepsin D. To address this hypothesis we focused on a defined feeding phase of a single tick species, namely partially engorged females of *I. ricinus*. Two-pronged profiling strategy involving biochemical assays and PCR-based cloning displayed a simultaneous expression and activity of the above listed peptidase types in the tick digestive tissue. An improved global insight increases the possibilities for practical interventions involving vaccines and offers a better understanding of vector-pathogen interactions at the primary interface, namely the tick gut.

## Results

### Functional profiling of multiple peptidase activities in the gut of *I. ricinus*

**Figure 1:**
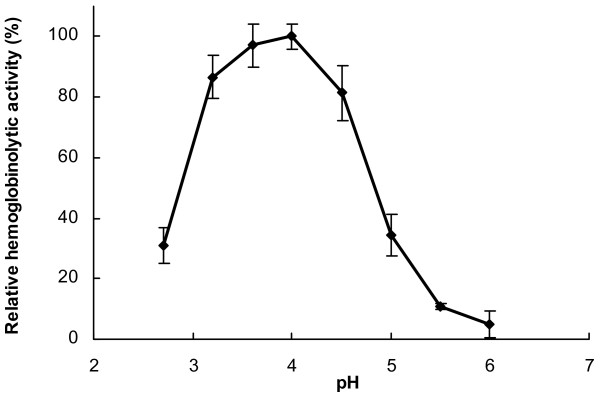
**The pH profile of hemoglobinolytic activity in the *I. ricinus *gut tissue extracts**. Gut tissue was dissected from partially engorged tick females (the 5-th day of feeding), washed from the gut contents and extracted. Fluorescently labeled AMC-hemoglobin was digested *in vitro *with gut extract at various pH values. The relative rate of degradation of the substrate was determined using the measurement of fluorescence in a continuous hemoglobinolytic assay. The error bars indicate standard deviations of the mean of triplicates.

Gut tissue extract prepared from partially engorged *I. ricinus *females (5th day of feeding) was tested on degradation of the biologically relevant substrate hemoglobin. Proteolysis was analyzed using a fluorescence assay incorporating AMC-hemoglobin. Optimal proteolysis was measured at acidic pH between 3.0 and 4.5 (Fig. 1). No substantial degradation occurred above pH 6.0. At optimum pH (~4.0), hemoglobinolytic activity was inhibited by the small molecule inhibitors E64 and pepstatin that selectively target cysteine and aspartic peptidases, respectively. A combined application of both compounds resulted in nearly complete blockage of the activity (~97% inhibition), the individual treatment showed about 80% inhibition (~83% and ~78% for E64 and pepstatin, respectively). In contrast, inhibitors of serine peptidases and metallopeptidases Pefabloc and EDTA, respectively, were ineffective (data not shown).

**Figure 2:**
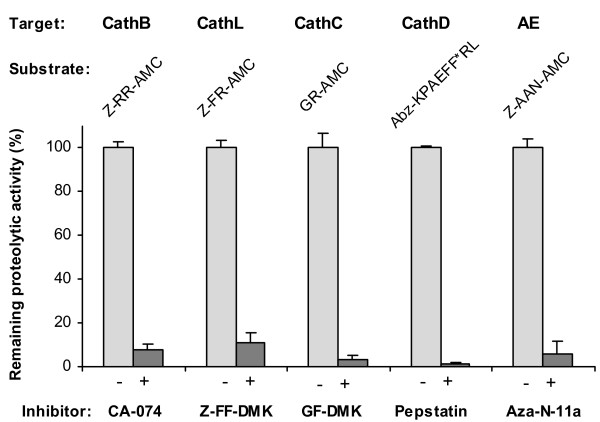
**Activity profiling of *I. ricinus *gut peptidases**. Peptidolytic activities in the gut tissue extract of partially engorged tick females (the 5-th day of feeding) were demonstrated *in vitro *with selective peptide substrates (structure of the fluorogenic substrates is indicated). The activity for individual substrates was suppressed in the presence of selective peptidase inhibitors to obtain diagnostic responses indicative of a protease type. Values are expressed as percent inhibition of the control activities. The identified major activities (Target) correspond to papain-type peptidases cathepsin B, L and C (CathB, CathL and CathC, respectively), cathepsin D-like aspartic peptidase (CathD) and asparaginyl endopeptidase (AE). The assay was performed at pH 4.0, an optimum pH for hemoglobin degradation by the gut extract. The activity of AE and CathL was measured in the presence of CA-074 inhibitor to prevent an interference with the activity of CathB. The error bars indicate standard deviations of the mean of triplicates.

Next, we focused on dissecting the major component peptidases in gut extracts responsible for the acidic degradation of blood meal using peptidase selective substrates and inhibitors (Fig. 2). At pH 4.0, hydrolytic activity cleaving the substrate Z-Arg-Arg-AMC (i.e., suggestive of cathepsin B activity) was inhibited 90% by the cathepsin B inhibitor, CA-074. Likewise, activity against Z-Phe-Arg-AMC was inhibited 90% by Z-Phe-Phe-DMK. Dipeptidyl peptidase activity of cathepsin C was measured with Gly-Arg-AMC and inhibited > 95% by Gly-Phe-DMK. Asparaginyl endopeptidase activity as measured with Z-Ala-Ala-Asn-AMC was inhibited > 95% by the azapeptide, Aza-N-11a. Cathepsin D-like activity measured with Abz-Lys-Pro-Ala-Glu-Phe-Nph-Ala-Leu was effectively inhibited (~98%) by pepstatin.

Thus, five significant endo- and exopeptidase activities of the cysteine and aspartic classes of peptidases were profiled in the gut tissue of *I. ricinus*. The identified activities include (i) the CA clan (papain-type) cysteine peptidases: cathepsins B, L, and C and a Clan CD asparaginyl endopeptidase (legumain), and (ii) a Clan AA aspartic peptidase activity: cathepsin D.

### Genetic screening of gut tissue identifies cDNAs encoding one aspartic and four cysteine peptidases

Single stranded cDNA derived from the gut of *I. ricinus *was used as a template for identification and cloning of genes encoding cysteine and aspartic peptidase precursors. Based on results of the functional screening of peptidase activities, multiple protein and cDNA alignments were performed to identify conserved domains in cathepsins B, L, D and C as shown for the schistosomal orthologues in Table 1. Degenerate primers derived from these motifs are listed together with expected PCR product lengths, optimal annealing temperatures, number of sequenced clones and number of identified isoforms (Table 1). Two isoforms of cathepsin B and one form each of cathepsins L, C and D were identified.

**Table 1:** Degenerate PCR primers used for identification of genes encoding gut-associated peptidases of *I. ricinus*

**peptidase**	**cathepsin B**	**cathepsin L**	**cathepsin C**	**cathepsin D**
***forward primer***	^5'^CKTGYGGDTCTTGTTGGG ^3'^	^5'^CAGTGCGGVTCCTGCTGGGC^3'^	^5'^GTCGACACKCCYGCBAACTGCAC^3'^	^5'^TCACCCCAGCCKNCGTTCCA^3'^
***reverse primer***	^5'^GGCTCCTCTAAYYTBTGGGT ^3'^	^5'^GGCGATTCCGCACTGGTTGT^3'^	^5'^CCRTAGWAACCWCCMACATA^3'^	^5'^GAACACGTCNCCBARDATCCA^3'^
Sm* GenBank	*CAC85211*	*CAA83538*	*CAA83543*	*AAB63442*
Sm* forward domain	^117^SSCGSCWA^124^	^123^LCGSCWA^129^	^20^ADTPANC^26^	^85^GSSNLWV^91^
Sm* reverse domain	^313^WNSDWGD^319^	^304^NMCGIA^309^	^337^YIGGYY^342^	^355^WILGDIF^361^
***annealing temperature***	55°C	53°C	62°C	53°C
***product length***	608 bp	554 bp	998 bp	855 bp
***sequenced clones***	12	8	8	8
***isoforms***	2	1	1	1

*- orthologues in *Schistosoma mansoni*

Hybridization screening of the gut cDNA library with radio-labeled PCR amplicons had been previously used to obtain the full-length coding sequence for IrAE [13]. Here the same approach succeeded in identifying full length cDNA sequences for cathepsins B, L and D. The full cDNA sequence of cathepsin C was generated by overlapping PCR, 5' and 3' RACE PCR fragments.

For the sake of consistency, we adopted a nomenclature for these enzymes previously used for schistosomal peptidases [15,18] – a nomenclature already applied to the *I. ricinus *asparaginyl endopeptidase (IrAE) [13]. Thus, we designated the novel peptidases as IrCB for cathepsin B (form 1), IrCL for cathepsin L, IrCC for cathepsin C and IrCD for cathepsin D.

**Figure 3:**
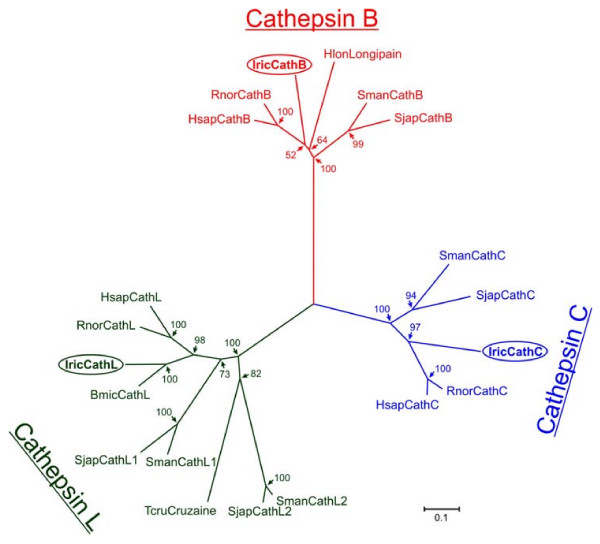
**Phylogenetic relation of *Ixodes ricinus *gut-associated cysteine peptidases (clan CA, family C1) to selected representatives of the papain family**. The tree was reconstructed by the Neighbor-joining method using amino acid sequences spanning across the homologous domains of mature enzymes. The horizontal bar represents a distance of 0.1 substitutions per site. Numbers at the branches display bootstrap support. **Cathepsins B**: *Homo sapiens *(AAH95408), *Rattus norvegicus *(P00787), *Ixodes ricinus *(ABO26563), *Haemaphysalis longicornis *(BAF43801), *Schistosoma mansoni *(P25792), *Schistosoma japonicum *(P43157); **Cathepsins L**: *H. sapiens *(M20496), *R. norvegicus *(AAH63175), *I. ricinus *(ABO26562), *Boophilus microplus *(AF227957), *S. mansoni *(CathL1 (Z32529), CathL2 (U07345)), *S. japonicum *(CathL1 (U38476), CathL2 (U38475)), *Trypanosoma cruzii *(P25779); **Cathepsins C**: *H. sapiens *(X87212), *R. norvegicus *(D90404), *I. ricinus *(ABV29335), *S. mansoni *(Z32531), *S. japonicum *(U77932).

The three clan CA cysteine peptidases, namely IrCB, IrCL and IrCC could be clearly classified using multiple sequence alignments followed by a phylogenetic analysis (Fig. 3). The GenBank blast program blastp [19] search of the IrCD sequence revealed the closest relation (55% identity) as longepsin, the aspartic peptidase from *H. longicornis *[12].

#### *I. ricinus *cathepsin B (IrCB)

**Figure 4:**
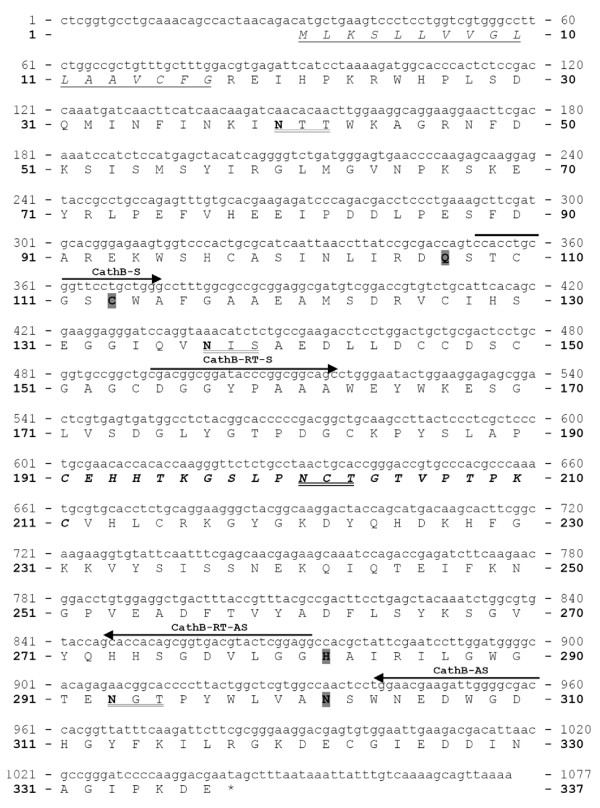
**Nucleotide and deduced amino acid sequence of *Ixodes ricinus *cathepsin B, form 1 (IrCB)**. The PCR primers used are upperlined and named as in Table 1 and 2. The predicted 17 AA signal peptide is underlined, in italics. Three potential *N*-glycosylation sites are double underlined and the respective asparagine residues are in bold; Active site residues C^113^, H^282^, Q^107^and N^302 ^are bold and shaded. Occluding loop responsible for cathepsin B exo-peptidase activity is bold, and italics.

The nucleotide and the deduced amino acid sequences of IrCB (form 1) enzyme precursor are shown in Fig. 4. The cDNA sequence [GenBank:EF428206] is 1073 bp long and contains one open reading frame encoding a polypeptide of 337 amino acid (AA) residues. Use of the SignalP 3.0 server [20] predicts a signal peptide cleavage between G^17 ^and R^18^. The pro-enzyme has a theoretical mass of 35.725 Da and an isoelectric point 5.76. The catalytic residues C^113^, H^282 ^and two other active site residues Q^107 ^and N^302 ^were found in positions typical for the C1 peptidase (papain) family. The protein has three potential *N*-glycosylation sites predicted by the NetNGlyc 1.0 Server [21]. The occluding loop responsible for the putative exo-peptidase (peptidyl dipeptidase) activity is predictable between C^191 ^and C^211^. Also, the domain ^297^YWLVANSWxxDWGD^310 ^accords to a domain previously described as being associated with the hemoglobinase activity of cathepsin B in blood feeding helminths [22].

#### *I. ricinus *cathepsin L (IrCL)

**Figure 5:**
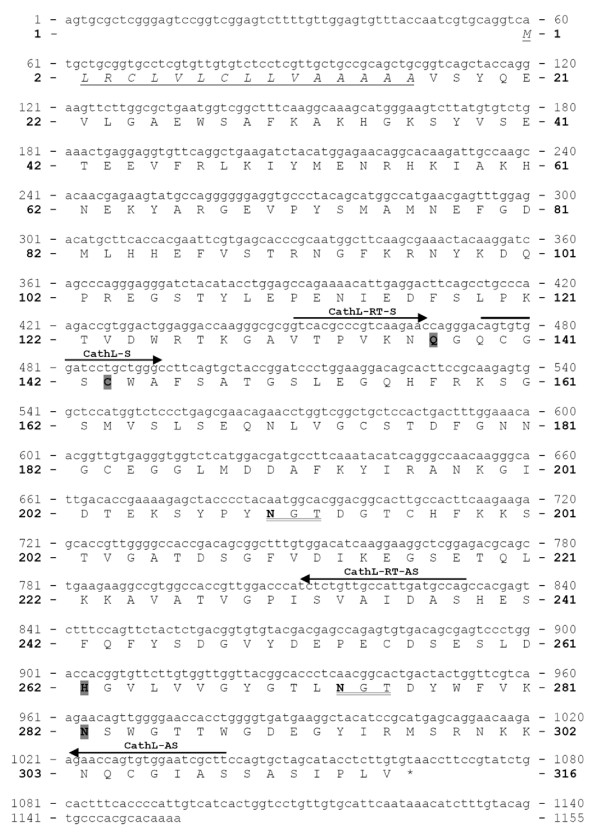
**Nucleotide and deduced amino acid sequence of *Ixodes ricinus *cathepsin L (IrCL)**. The depictions of PCR primers, putative 16 AA signal peptide, two potential *N*-glycosylation sites and active site residues C^143^, H^262^, Q^137 ^and N^282 ^are as in Fig 4.

Cathepsin L is another member of the papain family of peptidases that we have identified from *I. ricinus *guts (Fig. 5). The cDNA sequence [GenBank:EF428205] is 1151 bp long and contains one open reading frame encoding an enzyme precursor of 316 AA residues. The C1 family active site residues of IrCL were identified as Q^137^, C^143^, H^262 ^and N^282^. Predicted signal peptide is 16 AA long and the molecular weight of the pro-enzyme without the signal peptide is 35.403 Da with a theoretical isoelectric point 5.79. The proenzyme has two potential *N*-glycosylation sites both within the mature peptidase.

#### *I. ricinus *cathepsin C (IrCC)

**Figure 6:**
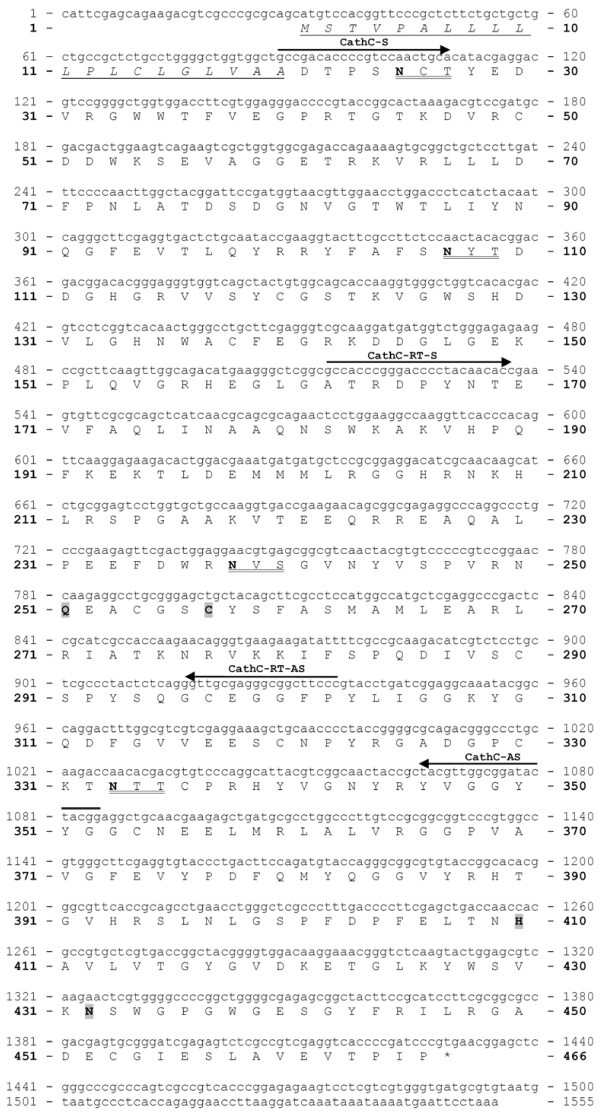
**Nucleotide and deduced amino acid sequence of *Ixodes ricinus *cathepsin C (IrCC)**. The depictions of PCR primers, putative 20 AA signal peptide, four potential *N*-glycosylation sites and active site residues C^256^, H^409^, Q^250 ^and N^431 ^are as in Fig 4.

Cathepsin C belongs to the clan CA papain family, but unlike B and L it acts solely as an exopeptidase. It has been shown to sequentially remove dipeptides from the substrate N-terminus (therefore alternatively named as dipeptidyl peptidase I). The cDNA sequence of IrCC [GenBank: EU128750] is 1597 bp long and encodes an enzyme precursor of 465 AA residues (Fig. 6). Calculated molecular weight of the proenzyme without signal peptide is 49.812 Da and the theoretical isoelectric point is 6.88. Based on the structure of human cathepsin C, the active site residues are Q^250^, C^256^, H^409 ^and N^431^. The predicted signal peptide counts for 20 AA residues and the proenzyme has four potential *N*-glycosylation sites, one close to the predicted mature N-terminus.

#### *I. ricinus *cathepsin D (IrCD)

**Figure 7:**
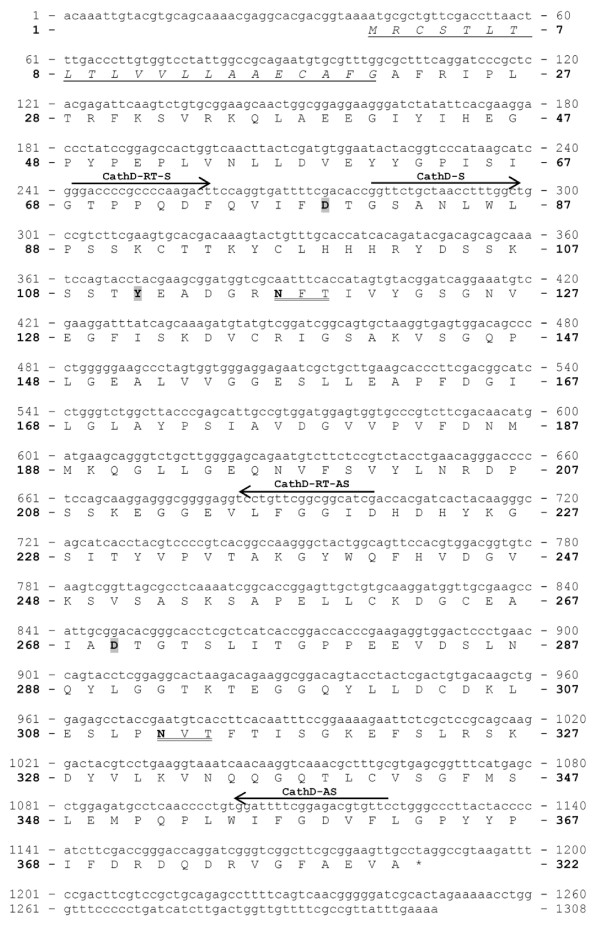
**Nucleotide and deduced amino acid sequence of *Ixodes ricinus *cathepsin D (IrCD)**. The depictions of PCR primers, putative 21 AA signal peptide, two potential *N*-glycosylation sites and active site residues D^79^, Y^111^, D^270 ^are as in Fig 4.

A cathepsin-D-like aspartic peptidase precursor of 322 amino acid residues (Fig. 7) is encoded by a 1304 bp long cDNA sequence [GenBank:EF428204]. The predicted signal peptide counts for 21 AA residues. The active site contains two catalytic aspartic acid residues D^79 ^and D^270 ^within the conserved D-T-G motifs and Y^111^. Molecular weight of the proenzyme without the signal peptide is 39.451 Da and the theoretical isoelectric point is 4.75. There are only two possible *N*-glycosylation sites in the IrCD proenzyme.

### Differential expression of peptidases during development and in tissues

**Figure 8:**
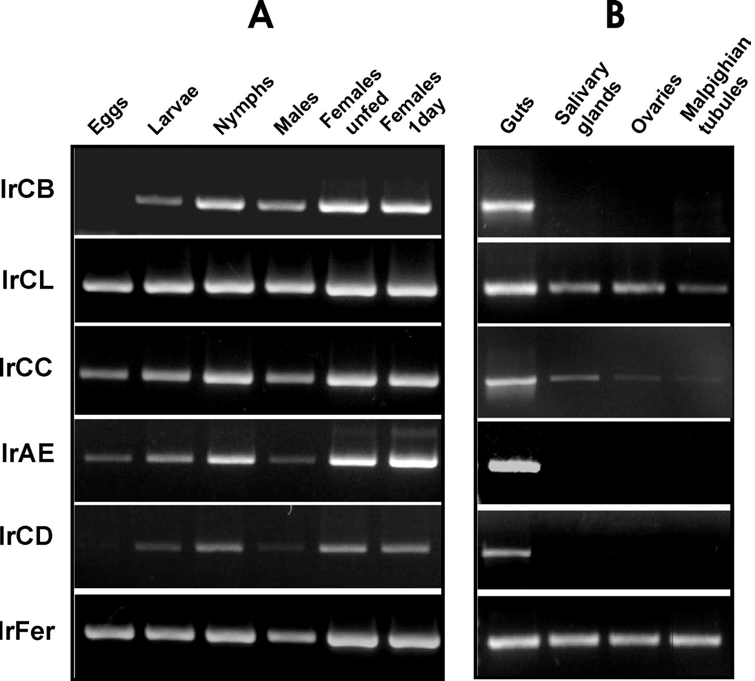
**Stage and tissue expression profiles of *Ixodes ricinus *cysteine and aspartic peptidases**. Messenger RNA levels of individual enzymes were determined by semi-quantitative two-step RT PCR. **Panel A**: Expression of peptidase mRNAs in whole body homogenates of *I. ricinus *eggs, unfed larvae, unfed nymphs, males, unfed females and females attached for 1 day on the guinea pigs. **Panel B**: Expression of peptidase mRNAs in tissues dissected from partially engorged females (the 5-th day of feeding). The abbreviations used are as in the text. IrFer shows the mRNA amplification of tick ferritin used as template loading control. (For details, see Methods).

Gene-specific PCR primer sets for the newly identified cDNAs and IrAE cDNA (Table 2) were used to amplify the relevant peptidase genes from different tick developmental stages and tissues. Semi-quantitative RT-PCR of whole-body homogenates revealed that IrCL, IrCC and IrAE are abundantly present in all developmental stages including eggs (Fig. 8A). In contrast, messages for IrCB and IrCD were absent from tick eggs. No apparent differences were observed for any enzyme expression between un-fed and freshly attached females suggesting that the enzyme messages are not changed in the initial feeding phase. Once partially engorged, it is possible to reliably dissect individual organs of females for RT-PCR tissue profiling (Fig. 8B) and the data demonstrate that all the peptidases of interest are co-expressed in the gut towards the end of the slow feeding period [7] what indicates their simultaneous action in a putative cascade or network. Moreover, IrCB, IrAE and IrCD seem to be strictly gut-specific, whereas messages for IrCL and IrCC were also found in other tick tissues. Negative controls in which the template cDNA was replaced by sterile distilled water gave no PCR products (data not shown).

**Table 2:** Gene specific primers used for RT-PCR expression profiling of *I. ricinus *peptidases

**peptidase**	**forward primer**	**reverse primer**	**annealing temperature**	**product length**
***IrAE***	^5'^TCGGTGACGCTGAGAAGACTGAA^3'^	^5'^TAGATTATGCCCGATGACTGTTGG^3'^	55°C	239 bp
***IrCB***	^5'^CGACGGCGGATACCCGGCGGCAGC^3'^	^5'^CCGAGTACGTCACCGCTGTGGTGC^3'^	60°C	360 bp
***IrCL***	^5'^GTCACGCCCGTCAAGAACC^3'^	^5'^TGGCATCAATGGCAACAGAGA^3'^	62°C	382 bp
***IrCC***	^5'^CCACCCGGGACCCCTACAACACC^3'^	^5'^GGAAGCCGCCCTCGCAACC^3'^	68°C	447 bp
***IrCD***	^5'^GGACCCCGCCCCAAGACT^3'^	^5'^CGATGCCGCCGAACAGGA^3'^	61°C	459 bp

## Discussion

The present functional and genetic profiling in the gut of the hard tick *I. ricinus *has identified a number of peptidase activities and genes. The overall goal was to have a better global understanding of the component peptidases for one important tick species in a particular feeding phase, namely partially engorged *I. ricinus *females, in contrast to the present fragmented picture regarding individual enzymes in a variety of different tick species. Also, this broad approach offers both the possibility to compare entire digestive systems with other hematophagous parasites as well as to investigate the potential of one or more component peptidases as molecular vaccines.

Initial activity profiling of *I. ricinus *gut extracts using a biologically relevant protein substrate (AMC hemoglobin) indicated that hemoglobinolysis is optimal at acid pH, a finding in accordance with data presented for other tick species [5,23,24]. This suggested that proteolysis is mediated by peptidases belonging to the aspartic and/or cysteine peptidase classes which are known to operate optimally at acid pH [25]. This conclusion was further supported by the sensitivity of hemoglobinolysis to class-selective peptidase inhibitors. Accordingly, we functionally scanned gut extracts for individual peptidase activities with a battery of diagnostic, small molecule substrates and inhibitors. These studies revealed the presence of four cysteine peptidase activities, cathepsins B, C, L and AE, and an aspartic peptidase activity, cathepsin D.

To identify the peptidase genes putatively responsible for the activities measured in gut extracts, we next screened gut-derived cDNA with degenerate primers designed to amplify individual peptidases. Five cysteine and one aspartic peptidases were classified: IrAE [13], IrCB, IrCL, IrCC and IrCD. Interestingly, from approximately 10 sequenced amplicons of each peptidase cDNA, only IrCB presented as two different isoforms. The finding is comparable with the data for hematophagous flukes, *Schistosoma mansoni *[26] and *Trichobilharzia regenti *[27], both of which have more than one cathepsin B isoforms.

Sequence comparison with human cathepsin B reveals that IrCB has the signature 'occluding loop' necessary for its exopeptidase (specifically, peptidyl dipeptidase) activity [28]. Regarding murine cathepsin C, this enzyme was previously shown to process and activate granulocyte serine peptidases by the removal of N-terminal dipeptides [29]. Thus, it would be of interest to test the potential competence of cathepsin C to processes cubulin-like serine peptidases inducing lysis of host blood cells [30]. The primary structure of the IrCD precursor is homologous to longepsin from *H. longicornis *[12] with two conserved Asp-Thr-Gly (DTG) catalytic site motifs either side of the substrate binding groove, a structure not shared by the more evolutionary distinct yolk-processing tick cathepsin D [31] and tick heme-binding aspartic peptidase [32] in the eggs of *B. microplus*. Finally, a search through the available EST database (NCBI Blast with a limitation to tick ESTs) indicated the existence of several isoforms for cathepsins B, L, AE and cathepsin D, but only one form of cathepsin C in the whole body derived cDNA of the closely related tick species, *Ixodes scapularis *[33]. Certainly, these preliminary data need to await final contig assembly and gene annotations and show the need for tick gut transcriptome projects.

With the exception of the eggs, host blood is taken up and processed to provide energy and nutrients for the transition from larva to nymph and finally, to adult male or female [4]. By RT-PCR, enzymes under study are expressed in all the feeding developmental stages indicating their simultaneous action in digestive cells. Notably, however, IrCB and IrCD are not expressed in eggs, suggesting a function specifically associated with blood digestion. In support of this notion, the tissue-specific RT-PCR demonstrated that both peptidases are expressed solely in the gut. Likewise, IrAE is also restricted to the gut but, being also found in eggs, must have an additional function(s) not associated with the blood meal.

## Conclusion

The combined biochemical and genetic analyses presented in this study demonstrate that *I. ricinus *expresses a suite of gut-associated cysteine and aspartic peptidases in order to catabolize ingested host proteins as a nutrient source. The data accord with previous results for enzyme activities in different tick species [5,24]. The particular combination of cysteine and aspartic peptidases comprising AE, and cathepsins B, C, D and L, operating at acidic pH and localized to the gut, is remarkably similar to those found in phylogenetically distant nematodes [17,34,35] and platyhelminths [15,16,34]. Indeed, cysteine and aspartic peptidases also contribute to amino acid acquisition in protozoa such as *Plasmodium *[36,37]. Therefore, and as noted by Delcroix et al. [16] for the platyhelminth *S. mansoni*, digestive systems based on cysteine and aspartic peptidases are widespread in invertebrates and stand in contrast to those systems utilizing serine peptidases (e.g., in insects and vertebrates). The present report extends this observation to include arthropods, specifically, ixodid ticks.

On an applied note, gut-associated peptidases may prove useful as vaccine targets. Other ixodid gut proteins, such as Bm86/Bm95 or Bm91, are suitable antigens for vaccination strategies (reviewed in de la Fuente and Kocan [38]). With this goal in mind, the detailed molecular and cellular characterization of the *I. ricinus *peptidases will be the subject of future reports.

## Methods

### Animals

*I. ricinus* ticks were collected by flagging in woodland localities around České Budějovice in the Czech Republic. Adult males and females were kept separately in glass vials in wet chambers with humidity of about 95% and temperature 26°C. If not stated otherwise, the females were allowed to feed naturally for 5 days on laboratory guinea pigs, carefully removed by forceps and referred to as partially engorged ticks in experiments described below. Laboratory animals were treated in accordance with the Animal Protection Law of the Czech Republic no. 246/1992 Sb.

### Materials

The 7-amino-4-methylcoumarin (AMC)-conjugated bovine hemoglobin was prepared according to Partanen et al. [39]. All peptidyl AMC-substrates were from Bachem, Abz-Lys-Pro-Ala-Glu-Phe-Nph-Ala-Leu (Abz, aminobenzoic acid; Nph, 4-nitrophenylalanine) substrate was prepared as described in Máša et al. [40]. Peptidase inhibitors were from Bachem, Gly-Phe-diazomethyl ketone (DMK) was prepared as described in Green and Shaw [41]. The aza-peptide Michael acceptor (CBz-Ala-Ala-(aza-Asn)-CH = CH-COOEt) further referred to as Aza-N-11a [42] was kindly donated by Dr. J.C. Powers of the School of Chemistry and Biochemistry, Georgia Institute of Technology, Atlanta, Georgia.

### Preparation of the tick gut tissue extract

For an experiment, tissues were dissected from 10 partially engorged *I. ricinus *females. The gut contents were carefully removed with a special care not to disrupt the epithelium. Cleaned guts were washed in phosphate-buffered saline solution (PBS) and pooled. The gut tissue extract (150 μg protein/ml) was prepared by homogenization of the gut tissue (without contents) in 1 ml of 0.1 M Na-acetate, pH 4.5, 1% CHAPS, 2.5 mM DTT using teflon-glass homogenizer on ice. The homogenate was centrifuged for 10 min at 10000 × g, the supernatant was filtered with Micropure-0.22 Separator (Millipore) and stored at -80°C.

### Quantification of hemoglobin degradation

Hemoglobinolytic activity was assayed using AMC-hemoglobin as a fluorogenic substrate [43]. Digestion of fluorogenic AMC-hemoglobin (0.5 μg supplemented with 2 μg of bovine hemoglobin) was performed at 35°C with the gut tissue extract (20-fold diluted stock solution) in 0.1 M Na-citrate-phosphate, pH 2.5–8.0 including 2.5 mM DTT, 25 mM NaCl and 0.05% Tween 20 in a reaction mixture of 100 μl. The proteolytic fragmentation of AMC-hemoglobin results in an increase of the fluorescence intensity that was continuously monitored to determine the relative reaction rate. The fluorescence signal was measured using a GENios Plus reader (TECAN) at 360 nm excitation and 465 nm emission wavelengths. For the hemoglobinolytic assay in the presence of a peptidase inhibitor, an aliquot of the extract was preincubated (15 min at 35°C) in the same buffer pH 4.0 with 10 μM E64, 10 μM pepstatin, 1 mM Pefabloc or 1 mM EDTA.

### Profiling component gut peptidases with substrates and inhibitors

Peptidase activities were identified and characterized by hydrolysis of the following fluorogenic substrates: 25 μM Z-Arg-Arg-AMC for cathepsin B [44], 25 μM Z-Phe-Arg-AMC for cathepsin L [44], 30 μM Gly-Arg-AMC for cathepsin C [45], 40 μM Abz-Lys-Pro-Ala-Glu-Phe-Nph-Ala-Leu for cathepsin D [40], and 30 μM Z-Ala-Ala-Asn-AMC for asparaginyl endopeptidase [46] as described previously [40,47]. The activity measurement was performed at 35°C using an aliquot of the gut tissue extract (20 to 200-fold diluted stock solution) in 0.1 M Na-acetate, pH 4.0 including 2.5 mM DTT (for cysteine peptidases) and 25 mM NaCl (for cathepsin C). For the activity assay in the presence of peptidase inhibitors, an aliquot of the extract was pre-incubated (15 min at 35°C) in the same buffer with the inhibitor: 10 μM CA-074 for cathepsin B [48], 10 μM Z-Phe-Phe-DMK for cathepsin L [49], 1 μM Gly-Phe-DMK for cathepsin C [41], 10 μM pepstatin for cathepsin D [50] or 1 μM Aza-N-11a for asparaginyl endopeptidase [42]. Hydrolytic activity was continuously measured after addition of substrate in a fluorescence reader GENios Plus at 320 nm excitation and 420 nm emission wavelengths (for Abz-containing substrate) or at 360 nm excitation and 465 nm emission wavelengths (for AMC-containing substrates). Assays of asparaginyl endopeptidase and cathepsin L were measured in the presence of 10 μM CA-074 to prevent confounding hydrolysis by cathepsin B [16,47].

### cDNA synthesis

Tissues (gut, salivary glands, ovaries and Malpighian tubules) were dissected from partially engorged females in a wax filled Petri dish with phosphate-buffered saline (PBS) under a binocular dissection microscope. The whole body homogenates from different developmental stages were prepared by crushing the appropriate number of eggs, larvae, nymphs, males, unfed females and females removed from guinea pigs one day after attachment using mortar and pestle and repeated freezing under liquid nitrogen. For total RNA isolation, the samples were further homogenized in a micro-tube with a plastic pestle in the TRI Reagent^® ^solution (Sigma) at 1 ml per 50–100 mg of wet tissue and processed according to the instructions provided with the TRI-reagent kit (Sigma). Isolated total RNA was stored at -80°C and further used for preparing single stranded cDNA templates using Superscript II (Invitrogen) and oligo(dT) primers, following the instructions provided by the manufacturer or for RT-PCR experiments described below.

### Designing PCR oligonuclotide primers

Degenerate primers were designed from conserved domains of *Schistosoma mansoni, S. japonicum*, mosquito, rat and human peptidases. Protein and nucleotide sequences downloaded from the NCBI GenBank web site were used for multiple ClustalW alignments in DNAstar MegAlign software (Lasergene). The oligonucleotides are listed in Table 1.

### PCR and rapid amplification of cDNA ends (5'- and 3-' RACE)

Mastercycler gradient (Eppendorf) was used to optimize PCR amplifications. Amplicons were purified, ligated into vector plasmid pCR 4-TOPO using the TOPO TA^® ^Cloning Kit (Invitrogen) and transformed into *E. coli *TOP 10 cells (Invitrogen). Clones containing ligated PCR products were sequenced using an automated sequencer model ABI Prism 3130 XL and the BigDye^® ^Terminator sequencing kit (Applied Biosystems) with appropriate sequencing primers. Sequence data were compared by blastn [51] against the NCBI GenBank database records. To obtain complete cDNA sequences, 3' RACE PCR was performed using a modified protocol for SMART™ cDNA Library Construction Kit (Clontech, BD Biosciences) described previously [52]. The N-terminal sequences including signal peptides and the 5' untranslated regions were determined using the Invitrogen 5'RACE system and instructions provided by the manufacturer.

### cDNA library construction and screening

The protocol for construction of the *I. ricinus *gut-derived cDNA library with SMART™ cDNA Library Construction Kit (Clontech, BD Biosciences) and the Gigapack^® ^III Gold packaging extract (Stratagene) as well as the method of cDNA library screening by [P^32^]dATP radio-labeled gene-specific probes have been described previously [13].

### Phylogenetic analysis

The primary sequences used for phylogenetic analysis comprised the conserved domains spanning across the mature enzyme sequences without pro-domains. Sequences were obtained from the MEROPS database [53] and aligned in the program ClustalX 1.81 [54]. The alignment was manually checked using the BioEdit program [55]. Tree reconstruction employed the Neighbor Joining (NJ) method [56] in the program MEGA 2.1 [57]. Nodal supports were calculated with 1000 replications.

### Semi-quantitative RT-PCR

Gene specific PCR primer pairs (listed in Table 2) were designed for each peptidase type with DNAstar PrimerSelect software (Lasergene). Two-step RT-PCR was performed using total RNA templates (prepared as described above; 50 ng/μl final concentration) and the Enhanced Avian HS RT-PCR Kit (Sigma) according to the protocol provided by the manufacturer. Amplification of the ferritin mRNA, previously shown to be presented in all tick tissues and expressed independently of feeding [58], was used as a loading control.

## Abbreviations

AA, amino acid; Abz, aminobenzoic acid; AMC, 7-amino-4-methyl-coumarin; bp, base pairs; DMK, Gly-Phe-diazomethyl ketone; DTT, dithiothreitol; EDTA, ethylenediaminetetraacetic acid; EST, expressed sequence tag; NCBI, National Center for Biotechnology Information; Nph, 4-nitrophenylalanine; PCR, polymerase chain reaction; RACE, rapid amplification of cDNA ends; RT-PCR, reverse transcription polymerase chain reaction.

## Authors' contributions

DS designed the degenerate primers, identified and isolated the genes described in this study from the cDNA library, and participated in all aspect of this manuscript including its conception and drafting. ZF was involved in cloning and sequencing of full length cDNAs by 5- and 3'- RACE PCR and conducted the RT-PCR expression profiling. MH and MM designed and performed the substrate/inhibitor based profiling and characterization of endogenous activities in the tick gut extract. OH contributed to the present manuscript by alignments and phylogenetic analyses. CRC interpreted the data in relation to the digestive system of flatworms and nematodes, included the evolutional aspect of this work and contributed to the manuscript drafting. PK and MM were responsible for the overall conception and coordination of the study and participated in drafting the manuscript. All authors have read and approved the final version of this manuscript.
